# Left Ventricular Hypertrophy in Pediatric Hypertension: A Mini Review

**DOI:** 10.3389/fped.2017.00101

**Published:** 2017-05-11

**Authors:** Robert P. Woroniecki, Andrew Kahnauth, Laurie E. Panesar, Katarina Supe-Markovina

**Affiliations:** ^1^Division of Pediatric Nephrology and Hypertension, Stony Brook Children’s Hospital, School of Medicine, Stony Brook, NY, USA; ^2^Stony Brook University, Stony Brook, NY, USA; ^3^Division of Pediatric Cardiology, Stony Brook Children’s Hospital, School of Medicine, Stony Brook, NY, USA

**Keywords:** left ventricular hypertrophy, LVMI, target organ damage, childhood hypertension, cardiac magnetic resonance, echocardiography

## Abstract

Adults with arterial hypertension (HTN) have stroke, myocardial infarction, end-stage renal disease (ESRD), or die at higher rates than those without. In children, HTN leads to target organ damage, which includes kidney, brain, eye, blood vessels, and heart, which precedes “hard outcomes” observed in adults. Left ventricular hypertrophy (LVH) or an anatomic and pathologic increase in left ventricular mass (LVM) in response to the HTN is a pediatric surrogate marker for HTN-induced morbidity and mortality in adults. This mini review discusses current definitions, clinically relevant methods of LVM measurements and normalization methods, its epidemiology, management, and issue of reversibility in children with HTN. Pediatric definition of LVH and abnormal LVM is not uniformed. With multiple definitions, prevalence of pediatric HTN-induced LVH is difficult to ascertain. In addition while in adults cardiac magnetic resonance imaging is considered “the gold standard” for LVM and LVH determination, pediatric data are limited to “special populations”: ESRD, transplant, and obese children. We summarize available data on pediatric LVH treatment and reversibility and offer future directions in addressing LVH in children with HTN.

## Introduction

Framingham Heart Study proved that adults with arterial hypertension (HTN) have increased mortality due to myocardial infarction and stroke compared to adults without HTN.^1^ These “hard outcomes” have not been clearly demonstrated in children with HTN. However, the Bogalusa Heart Study showed that children with HTN become hypertensive adults and that the major etiologies of adult heart disease (atherosclerosis, coronary heart disease, essential HTN) begin by 5–8 years of age.^2^ Since there are no “hard outcomes” of the deleterious effects of HTN in children, they are assessed by the “end organ” or “target organ” damage (TOD). Pediatric HTN-induced TOD manifests as an injury to several organs: (A) kidney: microalbuminuria/proteinuria, chronic kidney disease (1, 2), (B) eye: retinopathy (3), (C) vessels: increase in intima media thickness, atherosclerosis, reduced arterial compliance (2), (D) brain: cognitive impairment (4), and (E) heart: left ventricular hypertrophy (LVH) (5). TOD associated with uncontrolled HTN leads overtime to “hard outcomes” observed later in life (6). However, if HTN is controlled at least some of the TOD has been shown to be reversible (1, 7). Therefore, it is of paramount importance for pediatricians to be able to recognize presence of TOD as early as possible to act on it and thus prevent one of the “hard outcomes” in adulthood. LVH independently of blood pressure (BP) levels predicted poor prognosis in adults enrolled in the Framingham Heart Study. The relative risk of cardiovascular mortality for every 50 g increment in left ventricular mass (LVM) was 1.73 in men and 2.12 in women (8). The focus of this mini review is on definition, detection, epidemiology, management, and reversibility of LVH.

## Definition

Left ventricular hypertrophy is defined as an increase in LVM in response to a disease state, due to either increase in left ventricular (LV) wall thickness or an increase in cavity size or both. These changes of LV diameters represent adaptive responses to a pathologic workload due to HTN or heart valve disease (as oppose to physiologic states of pregnancy or exercise/cardio-workout). However, LVH could also be related to infiltrative diseases of the myocardium or certain genetic disorders. HTN-induced LVH in children usually presents with an increase in wall thickness (concentric hypertrophy), without an increase in cavity size (eccentric hypertophy). Concentric LVH induced by HTN leads with time to LV dilatation, which results in a decline of the LV ejection fraction and eventually in “dilated cardiac failure” (9). Widening of the LV chamber creates circulatory difficulties and presents a vicious cycle to the cardiac muscle, increasing risk for cardiovascular complications (10). Both casual office BP readings and 24-h ambulatory blood pressure monitoring (ABPM) have a direct relationship to LVM/LVH: the higher office BP, the higher average BP recorded over the course of the day and night, or the higher ABPM BP loads (prevalence of abnormal elevated BP readings) the greater possibility for LVH (11). It is worth noting that long-standing white-coat HTN (normal ABMP and abnormal office BP) is associated with increased risks of cardiovascular complications in adults (12). However, this relationship is not consistent in number of pediatric patients with white-coat HTN (13). In summary, the definition of pediatric HTN-induced LVH pivots on an accurate assessment of LVM and determination of what normal versus abnormal is for a given individual. This in turn depends on the accuracy of LVM measurements, and LVH detection and normalization method used.

## Detection Methods

### Cardiac Magnetic Resonance (CMR)

Cardiac magnetic resonance uses strong magnetic fields that stimulate the hydrogen nuclei (present in water or fat) to release radio waves that can be interpreted by computerized scanners and generate images of the heart (14). In adults, CMR imaging is considered as the gold standard for assessment of LVM and LVH (15, 16). In addition, CMR estimates of LVM have been shown to be closely correlated to actual heart weight determined at autopsy in both animal and human models (16). CMR yields high-quality images across the entire left ventricle that is not impeded by thoracic fat deposits and chest wall expansion. Furthermore, CMR is used in the evaluation of congenital heart disease, ant its use has been recently reported in children and young adults on maintenance dialysis (17, 18), and renal transplant recipients (19). Our group has described the utility of CMR in overweight hypertensive children and reported reproducibility (20). However, CMR is not yet widely available, is more expensive than echocardiography (ECHO), has disadvantages such as claustrophobia and the need for sedation in small children, and the data on its utilization in general pediatric population with HTN are lacking. Abnormal LVM and LVH in pediatric CMR is defined as a *z* score greater than +2.0 utilizing published review that calculated and tabulated pooled weighted mean values that are specific for age and sex (21).

### Echocardiography

Although LVM determined by CMR is more accurate and reproducible, ECHO has lower cost and is a more accessible test compared with CMR. ECHO is an imaging technique that creates pictures of the heart utilizing high-frequency ultrasound waves. Whether it be two-dimensional, three-dimensional, or M-mode, ECHO is used to assess TOD and measure LVM. Echocardiographic studies determine the myocardial volume by subtracting the LV cavity volume from the volume of the correspondent epicardium. Upon obtaining the myocardial volume, multiplication by the myocardial density results in the LVM (22). The LVM can then be indexed to body surface area (BSA), or height^2.7^ to determine LVH (23). One of the challenges when using echocardiographic techniques to determine LVH is precisely finding the boundary between the cardiac blood pool and the endocardium (23). If this step was inaccurate due to, for example, poor acoustic window, or abundant chest fat tissue, there would be improper readings of the LV cavity volume and the epicardial volume. This in turn would result in inaccurate myocardial volumes when performing calculations and thus, inaccurate LVM levels and LVH indicators. For adults, a LVM index ≥51 g/m^2.7^ is used to define LVH based on a study by de Simone et al., which showed LVMI above this threshold is associated with more than four times increased risk of morbidity and mortality (24). The Fourth Report selected 51 g/m^2.7^ as their LVMI limit value to define LVH in children (25). However, this value does not adjust for growth and other potentially confounding factors. The Bogalusa Heart Study demonstrated that somatic growth is the strongest predictor of LVM (26). Therefore, LVM must be indexed to normalize the relationship without disregarding obesity. Foster et al. showed that normalizing LVM to BSA or height results in either underestimation or overestimation of LVM, respectively (27). They proposed lean body mass (LBM) as the ideal scaling variable for normalization. Although LBM can be measured by dual-energy X-ray absorptiometry, it is clinically difficult to ascertain (27). Foster et al. used LBM predictive equations and generated sex-specific LVM-for-LBM centile curves for children 5–18 years of age and defined LVH as LVMI-for-age >95th percentile (27). Despite this, most pediatric nephrologists index LVM to height^2.7^. Khoury et al. developed age- and sex-based LVMI (height^2.7^) centiles in 2009 (28). They observed little variation beyond age 9, suggesting their reference tables would only be needed for younger children. They defined LVH as LVM/height^2.7^ greater than 95th percentile for sex and age (28). According to their calculations after age 9 years, a constant 95th percentile value of 40 g/m^2.7^ (female), and 45 g/m^2.7^ (male) defines LVH (28). At present, it is challenging to say which indexing method is better because there is no one method without substantial limitations. Furthermore, ECHO cannot distinguish small but clinically significant changes in diastolic wall thickness from measurement error in individual children, even when measured by the same observer (29). Three-dimensional ECHO has also been utilized to quantify LVM and allows for LVM quantification using principles similar to CMR. LVM is determined by taking the difference between epicardial and endocardial volumes and may better account for ventricular morphology. Quantification of LVM by three-dimensional ECHO has been shown to be of use in the adult population; however, its use remains limited in pediatrics at this time (30).

### Electrocardiography (ECG)

There is no explicit ECG pattern predictive of abnormal LVM. Instead, there are host of electrical abnormalities (voltage and non-voltage criteria) that are associated with LVH. The most commonly used are the Sokolow–Lyon criteria (31), and voltage criteria must be accompanied by non-voltage criteria to be considered diagnostic of LVH (32). However, HTN-induced LVH could be easily misclassified by using ECG; therefore, ECG should not be used alone in determining presence or absence of LVH. However, ECG still has a place in TOD assessment as it gives independent information on the cardiovascular risk even after adjusting for LVM (32). In children with HTN, although ECG has high specificity (>90%), it is a poor screening test with low sensitivity (<35%) in evaluation of abnormal LVM (33).

Left ventricular hypertrophy detection methods summary is presented in Table 1.

**Table 1:** **Summary of pediatric LVH clinical detection methods**.

Method	Advantage	Limitation
ECG	– Availability (office/emergency room) – Low cost – Minimal time requirement – No need for sedation in small children – High specificity (>90%)	– Poor sensitivity (<35%) – No explicit ECG pattern predictive of LVH
ECHO M-mode	– 1st method of ECHO validated – Relatively simple and quick – Cost efficient – Superior endocardial definition – No need for sedation in small children	– Measures LV in one dimension – Assumes an ellipsoid LV shape with a ratio of long:short axis lengths of 2:1 – Relies on linear measurements of LV thickness, which, when cubed, increase the SD by a factor of 2–3× – Debated accuracy and reproducibility
ECHO 2D	– More accurate and reproducible than the M-mode method – Relatively simple and quick – Cost efficient – No need for sedation in small children	– Assumes an ellipsoid LV shape and a uniform LV wall thickness – Prone to error mathematical formulae to estimate the LVM (without cube function = less error than M-mode)
ECHO 3D	– Improved the intra- and interobserver variability as compared to M-mode and 2D ECHO – Does not rely on geometrical assumptions for calculating LVM – Correlates well with CMR	– More complicated and expensive equipment – Superior-quality 3D images are dependent on optimal echo windows (impaired in obese subjects) – Lack of pediatric data
CMR	– “Gold standard” in selected populations – Excellent the intra- and interobserver variability – Independent chest wall thickness	– Requires sedation in small children – Not widely available – Lack of pediatric data in general population – More expensive than ECHO

*ECG, electrocardiography; ECHO, echocardiography; M-mode, motion (over time) mode; 2D, two-dimensional ECHO; 3D, three-dimensional ECHO; CMR, cardiac magnetic resonance; LVH, left ventricular hypertrophy; LVM, left ventricular mass*.

### Epidemiology

The prevalence of LVH in children and adolescents is not precisely known partly because multiple definitions in different population exist. It varies from 4.8 to 50% in children with primary HTN (27, 28, 34–36) and has been reported as high as 55% in children after renal transplant (37) and 85% in children on dialysis (38). In children with chronic kidney disease, the following factors contribute to abnormalities in calculating LVM: male sex, higher body mass index, degree of anemia, fluid overload, and low-grade inflammation and can be more relevant than the effect of HTN (39).

In pediatric essential HTN, severe LVH defined as LVMI above 51 g/height^2.7^ has been found in 10–15% (34, 40, 41). The prevalence of LVH in children without HTN is unknown. In adults, it has been reported that obesity had significant independent associations with LVM and wall thickness (42), and this relationship has been confirmed in obese children and adolescents with normal office BPs (43, 44). In addition to body mass index, ethnicity contributes to differences in prevalence of LVH. A collaborative study of the International Pediatric Hypertension Association found that LVH and concentric hypertrophy occurred most frequently in hypertensive Hispanic children (35). In adults, LVH is more common in hypertensive African-Americans (AA) (50%) than in whites (33%) (45), and the adjusted risk of having LVH, whether indexed by height^2.7^ or by BSA, is greater for AA than for whites (46). In AA children with HTN, LVH prevalence was 49 versus 30% among non-AA (*p* < 0.05) (36). Children of AA descent with chronic kidney disease and high-risk *APOL1* genotypes have both higher prevalence of LVH (53 versus 12%, *p* < 0.01) and obesity (48 versus 19%, *p* = 0.01) than their non-AA counterparts (47). Women were thought to have a lower prevalence of LVH than men for any given level of BP (8). However, LVH prevalence differences between sexes are dependent on LVM normalized to either height, height^2.7^, or BSA. LVM was identical in men and women when using LV mass/fat-free mass as a partition value of 4.1 g/kg (48). Since high dietary intake of sodium is associated with increase in BP and elevated BP is associated with LVH (49), salt restriction is associated with decrease in the incidence of LVH (50). In children with chronic kidney disease, differences in LVH prevalence between girls and boys were also dependent on LVH definition. When LVH was defined by LVM indexed to height, girls had higher prevalence of LVH (16 versus 9%, *p* = 0.01); when LVH was defined by LVM relative to estimated LBM, prevalence of LVH was similar between girls and boys (18 versus 17%, *p* = 0.92) (51). It has been known that adults with diabetes mellitus have higher LV mass, independent of HTN (52, 53). Adult type II diabetics have significantly higher prevalence of LVH in hypertensive women (54). Similarly, diabetic children have significant changes in LV dimensions, and girls are more affected than boys (55). Finally, there is strong evidence for the genetic influence on LVH prevalence across several study designs, datasets and ethnicities (56–60), and several genetic conditions, i.e., hypertrophic cardiomyopathy, caused by mutations in sarcomere genes (61), and RASopathies are causally associated with LVH (62). However, specific genetic influences on prevalence of HTN-associated LVH are currently unknown.

### Management and Reversibility

Hypertension is a rare cause of heart failure in children and adolescents. In children, the rationale to treat LVH is based on adult data. Special attention should be given to children and adolescents with HTN and the following conditions: end-stage renal failure, diabetes, coarctation of the aorta, or Kawasaki disease. These patients are at increased risk of early cardiovascular events and heart failure (63). Non-pharmacologic hypertensive treatments such as dietary salt restriction, weight reduction, avoidance of smoking, and aerobic exercise have been effective in lowering cardiovascular risk factors in adults (64). In children with HTN, if BP does not improve by lifestyle modification or there is evidence of TOD, then pharmacologic treatment is indicated (65). In children with HTN, presence of LVH prompts treatment and LVH reversal becomes a major treatment goal. The baseline severity of LVH, and the degree and duration of HTN control determines slope of LVH reversal (40, 66, 67). In adults, any BP lowering agent lowers the risk of LVH (68). All anti-HTN classes are effective in lowering of BP in children; however, their differential effects on LVH have not been demonstrated in pediatric population (25). Angiotensin-converting enzyme (ACE) inhibitors and angiotensin receptor blockers (ARB) are especially effective in the regression of LVH due to host of additional BP-independent mechanisms: reduction of growth factors (TGF-beta), free oxygen radicals, inhibitory effects on myocyte growth, and collagen formation. In adults, treatment with ACE inhibitors or ARB increases insulin sensitivity (69, 70), whereas treatment with thiazides decreases insulin sensitivity (71, 72). In fact, ACE/ARB has been the drug of choice for over a decade for pediatric nephrologists who treat HTN (73). This preference comes not from pediatric data but from adult studies showing their beneficial, cardio and reno-protective effects in diabetes, HTN, and microalbuminuria (71, 72). In adults with HTN or with early-stage CKD, the addition of spironolactone to ACE/ARB reduced LVM and improved arterial stiffness (74). Spironolactone is usually used in children with congestive heart failure or as potassium sparring diuretic and not as first or second agent in HTN-induced LVH treatment. In adults, calcium channel antagonists are as effective as ACE inhibitors (such as Lisinopril) in their ability to reduce LVH and cardiac wall thickness (75). This is likely due to dominant effect of BP lowering over BP-independent effects. Another class of pharmacological agents that assist in the regression of LVH are beta receptor-blocking agents: both B1 receptor specific or non-selective that work through decreasing cardiac output and easing afterload facilitating LV remodeling. However, abnormalities in diastolic function associated with HTN-induced LVH are not reduced by reversal of LVH induced by antihypertensive treatment with beta-blockers (76). It is very interesting that in some children with primary HTN the main determinant of LVH regression is decrease in abdominal obesity with an increase in LBM rather than BP lowering (77). This conforms to recent adult study finding that higher BMI is associated with less reduction of hypertensive LVH: independent of BP control and of types of antihypertensive treatment (78). Although different classes of antihypertensive have different effects on LV mass in adults, the data regarding the specific effects of antihypertensive therapy on LVH in children are not adequate.

### Future Directions

Left ventricular mass and LVH are associated with HTN and with increased BMI. Efforts to diminish pediatric cardiovascular risk factors and prevalence of HTN-induced LVH should thus be connected to the public campaign to reduce obesity and increase childhood physical activity. In addition, emergence of effective approaches to issue of medication adherence may result in better long-term BP control. Recognition of food addictions, full understanding of sugar and food additives effects on anxiety, depression, BP, and hyperactivity may lead to effective interventions of addressing the root cause of obesity/HTN, which in turn may result with time in a decrease in the prevalence of LVH. Increased use of advanced technologies such as CMR or multidimensional ECHO should lead to improved detection of LVH and better understanding of its pathophysiology and epidemiology. Our improved understanding of cardiac electrogenesis and electrical remodeling in TOD may allow for more targeted treatment of electrical abnormalities associated with HTN. Furthermore, randomized controlled studies in pediatric HTN populations are needed to answer the question if available adult data can really be translated to children.

## Author Contributions

RW wrote the mini review and contributed to review of literature and design of the review. AK, LP, and KS-M contributed to review of literature and wrote the part of the review.

## Conflict of Interest Statement

The authors declare that the research was conducted in the absence of any commercial or financial relationships that could be construed as a potential conflict of interest.

## Footnotes

^1^www.framinghamheartstudy.org.

^2^www.clersite.org/bogalusaheartstudy/.
